# Altered Brain Functional Connectivity at Resting-State in Patients With Non-arteritic Anterior Ischemic Optic Neuropathy

**DOI:** 10.3389/fnins.2021.712256

**Published:** 2021-10-01

**Authors:** Pengbo Zhao, Han Lv, Pengde Guo, Yan Su, Ming Liu, Yan Wang, Haiqin Hua, Shaohong Kang

**Affiliations:** ^1^Department of Ophthalmology, Dongfang Hospital, Beijing University of Chinese Medicine, Beijing, China; ^2^Department of Radiology, Beijing Friendship Hospital, Capital Medical University, Beijing, China; ^3^Department of Radiology, Dongfang Hospital, Beijing University of Chinese Medicine, Beijing, China

**Keywords:** non-arteritic anterior ischemic optic neuropathy, functional connectivity, resting state, functional magnetic resonance imaging, neural plasticity

## Abstract

**Purpose:** To investigate the possible changes in functional connectivity (FC) in patients with non-arteritic anterior ischemic optic neuropathy (NAION) using resting-state functional MRI (fMRI).

**Methods:** Thirty-one NAION patients and 31 healthy controls were recruited and underwent resting-state fMRI scans. Regions of interest (ROIs) were defined as bilateral Brodmann’s area 17 (BA17). FC analysis was performed between the ROIs and the rest of the brain regions, and the between group comparisons of FC were performed. We conducted correlation analysis between the FC changes and the clinical variables in NAION patients.

**Results:** Compared with healthy controls, patients with NAION showed significantly decreased FC between the left BA17 and the right inferior frontal gyrus, left caudate nucleus. As for the right BA17, patients exhibited significantly increased FC with the left olfactory gyrus and decreased FC with the right superior frontal gyrus (SFG), right insula. Moreover, FC values between the right insula and the right BA17 were positively correlated with the right side of mean sensitivity in the central visual field (*r* = 0.52, *P* < 0.01) and negatively correlated with the right side of mean defect in the central visual field (*r* = −0.55, *P* < 0.01).

**Conclusion:** Our study indicated that patients with NAION showed significantly abnormal functional reorganization between the primary visual cortex and several other brain regions not directly related to visual function, which supports that NAION may not only be an ophthalmic disease but also a neuro-ophthalmological disease.

## Introduction

Non-arteritic anterior ischemic optic neuropathy (NAION) is the second most common type of optic neuropathy, with an annual incidence of 2.3–10.2 cases per 100,000 people in the United States (Johnson and Arnold, 1994; Hattenhauer et al., 1997) and 6.25 cases per 100,000 people in China (Xu et al., 2007). Typically, NAION is characterized by sudden, painless unilateral loss of vision (Miller and Arnold, 2015). Although the pathogenesis and pathophysiology of NAION remain undetermined, the lesions are thought to result from infarction of the short posterior ciliary arteries (vessels that supply the anterior portion of the optic nerve head) (Knox et al., 2000), which results in degeneration of retinal ganglion cells, followed by loss of their axonal structure (Zhang et al., 2010).

Previous studies of NAION have mainly concentrated on its pathophysiology, clinical characteristics, prevention, and treatment (Katz and Trobe, 2015; Qin et al., 2015; Berry et al., 2017; Keren et al., 2017). In general, studies concerning the structural and functional plasticity of the brain in cases of NAION are rare because conventional magnetic resonance imaging (MRI) provides little information pertaining to the lesion. Functional MRI (fMRI), a non-invasive neuroimaging technique, has made it possible to explore neural plasticity in NAION. The fMRI has been widely used to study changes in brain function in several eye-related diseases (Burton et al., 2004; Wang et al., 2008, 2021; Shao et al., 2015; Li et al., 2016; van Kemenade et al., 2017; Huang et al., 2018; Min et al., 2018; Xu et al., 2019). Additionally, several fMRI studies have demonstrated abnormal spontaneous brain activity in patients with NAION (Aguirregomozcorta et al., 2011; Guo et al., 2019, 2020).

Functional connectivity (FC) measures the degree of synchrony of the BOLD time-series between different brain regions (Tononi et al., 1994; Liu et al., 2015, 2017; Lv et al., 2018). However, few studies have provided information regarding the FC changes in NAION patients. The primary visual cortex is the first step in cortical visual processing, and its degree of activation is closely related to optic nerve damage that occurs in eye-related diseases. Previous studies have found FC alterations within the primary visual cortex in cases of primary angle-closure glaucoma (Wang et al., 2021), strabismus (Zhu et al., 2018), and amblyopia (Dai et al., 2019). In addition, another study has demonstrated that NAION not only damages the retinal ganglion cells and reduces optic nerve integrity, but also damages the visual cortex (Wang et al., 2011). A previous fMRI study found that activation in the bilateral occipital cortex was decreased after stimulating the affected eye in patients with NAION than after stimulating the eyes of healthy controls (Aguirregomozcorta et al., 2011). These findings indicate that NAION might be related to functional changes within the primary visual cortex. However, it remains unclear whether there are FC changes between the primary visual cortex and the other cortical regions in NAION. Here, we hypothesized that there may be significant changes in FC between the primary visual cortex and other cortical regions in NAION, and the changes would be consistent with the visual network pathology observed in patients with NAION.

## Materials and Methods

### Participants

Thirty-one patients with NAION (20 males and 11 females) who visited the ophthalmology Department at Dong Fang Hospital affiliated with Beijing University of Chinese Medicine were enrolled in the study according to the following inclusion criteria: (1) a typical clinical history of sudden, painless, and monocular visual loss or successive bilateral visual loss; (2) receival of standardized treatment and evaluation at our hospital; and (3) no history of coronary artery disease, hypertension, sleep disorders, or drug addiction. The exclusion criteria were as follows: (1) systemic features suggesting optic neuritis, giant cell arteritis, posterior ischemic optic neuropathy, or a history of optic tumor or other ocular disease; (2) symptoms of neurological disorders, mental disorders, or the inability or unwillingness to cooperate; and (3) abnormal function in the liver or kidney. In addition, 31 healthy controls (HCs) matched for age and gender were recruited according to the following criteria: (1) no history of ocular disease or symptoms of neurological disease; and (2) visual acuity > 1.0 on the vision chart. All participants underwent a vision acuity test, intraocular pressure measurement, a central visual field test, optical coherence tomography to measure retinal nerve-fiber layer thickness, and MRI scanning.

### Imaging Data Acquisition

All participants were scanned by a 1.5 Tesla MRI scanner (Intera Achieva System, Royal Philips, Amsterdam, Netherlands) with an eight-channel head coil. The participants were asked to wear sponge earplugs and a black blinder and to refrain from thinking about anything during the scans. The functional data were obtained using an echo planar imaging (EPI) pulse sequence with each scan. Thirty-five axial slices were acquired with the following parameters: repetition time = 3,000 ms, echo time = 30 ms, flip angle = 90°, field of view = 220 mm × 220 mm, matrix = 64 × 64, thickness = 3.6 mm, and gap = 0.72 mm, 100 time points. The total scan time was 300 s. Furthermore, high-resolution structural images (3D BRAVO) were acquired with the following parameters: matrix = 256 × 256, field of view = 256 mm × 256 mm, thickness = 1.0 mm, number of excitations = 2, repetition time = 6.5 ms, echo time = 3.2 ms, and flip angle = 8°, number of slices = 160.

### Functional Magnetic Resonance Imaging Data Processing

All data were analyzed using the Data Processing Assistant for Resting State fMRI (DPARSF)^1^, which is based on Statistical Parametric Mapping version 8 (SPM8)^2^ and the Resting-State fMRI Data Analysis Toolkit (REST)^3^, and was implemented in MATLAB 2014a (Mathworks, Natick, MA, United States). The following preprocessing steps were employed: the first 10 volumes were removed because of signal equilibrium and participants take time to adapt to the scanning environment; after that, slice timing and head motion correction were performed. Participants with head movements greater than 1.5 mm along any axis (x, y, or z) or greater than 1.5° in any direction were excluded (four patients were removed from data analysis for this reason). Next, based on the standard stereotaxic coordinate system, the corrected fMRI images were spatially normalized to a Montreal Neurological Institute (MNI) template brain; each voxel was resampled to isotropic 3 mm × 3 mm × 3 mm. The covariates (whole-brain head motion parameters, cerebrospinal fluid signal, and white matter signal) were removed after that. Then, linear trends in the time courses were removed and temporally bandpass filtered (0.01–0.08 Hz) to reduce the effect of physiological high-frequency respiration and cardiac noise, and low-frequency drift; finally, the images were smoothed with a full-width-at-half-maximum Gaussian kernel of 4 mm × 4 mm × 4 mm.

The primary visual cortex, known as Brodmann’s area 17 (BA17), is the core area of visual processing in the brain. A previous study reported that the activation in bilateral primary visual cortex was altered in patients with NAION (Aguirregomozcorta et al., 2011). Therefore, we defined the region of interest (ROI) as bilateral BA17 according to the WFU-atlas (Maldjian et al., 2003, 2004) and previous studies (Ding et al., 2013; Zhu et al., 2018). Each ROI was a sphere with a radius of 5 mm. The FC value was determined by the Pearson’s correlation coefficient of the time series of each ROI and other gray matter voxels. To improve the normal distribution, the correlation coefficients of r values were converted to z values by applying Fisher’s r-to-z conversion. The final fMRI results were presented by REST software and BrainNet Viewer.^4^

### Statistical Analysis

Two-sample *t*-tests were used to detect the differences in FC values between the two groups of participants, with gender, age, and duration of disease as covariates (*P* < 0.05, corrected for multiple comparisons using a false discovery rate).

Independent-sample *t*-tests were used to compare the clinical data between patients with NAION and HCs using SPSS 17.0 software (SPSS Inc., Chicago, IL) (*P* < 0.05, uncorrected). Pearson’s linear correlation analyses were used to assess the relationships between the mean FC values of brain regions with statistical difference and clinical parameters in NAION patients (*P* < 0.05, uncorrected). Furthermore, the receiver operating characteristic (ROC) curve method was performed to classify different FC values between NAION patients and HCs (*P* < 0.05, uncorrected).

## Results

### Demographic and Clinical Data

Table 1 shows the demographic and clinical data for the NAION patients and HCs. We found significant differences in vision acuity (*P* < 0.01), thickness of the retinal nerve-fiber layer (*P* < 0.01), intraocular pressure (*P* < 0.01), and the size of the central visual field (*P* < 0.01). No significant differences were found between groups in participant age.

**TABLE 1:** Demographic and clinical characteristics of the NAION patients and HCs.

	**NAION (*n* = 31)**	**HCs (*n* = 31)**	***t*-value**	***P*-value**
Age, years	52.74 ± 11.29	50.97 ± 8.20	0.71	0.48
Gender, male/female	20/11	20/11	NA	NA
Duration of illness (years)	6.00 ± 1.12	NA	NA	NA
VA-right	0.53 ± 0.40	1.08 ± 0.17	–7.14	0.00
VA-left	0.57 ± 0.40	1.10 ± 0.17	–6.75	0.00
RNFL-right (μm)	73.00 ± 23.25	97.58 ± 8.24	–5.55	0.00
RNFL-left (μm)	79.03 ± 28.51	97.80 ± 6.91	–3.56	0.00
IOP-right	14.61 ± 2.38	13.10 ± 1.27	3.16	0.02
IOP-left	15.35 ± 2.03	13.68 ± 1.62	3.60	0.00
**CVF**				
MD-right	12.76 ± 9.30	0.86 ± 1.33	7.05	0.00
MD-left	10.47 ± 9.05	0.92 ± 1.28	5.82	0.00
MS-right	13.83 ± 9.23	26.96 ± 1.42	–7.83	0.00
MS-left	16.79 ± 9.04	26.89 ± 1.29	–6.16	0.00

*NAION, non-arteritic anterior ischemic optic neuropathy; HCs, healthy controls; NA, not applicable; VA, vision acuity; RNFL, retinal nerve fiber layer thickness; IOP, intraocular pressure; CVF, central vision field; MD, mean defect; MS, mean sensitivity.*

### The Brain Areas With Functional Connectivity Differences Between the Non-arteritic Anterior Ischemic Optic Neuropathy Patients and Healthy Controls

**FIGURE 1:**
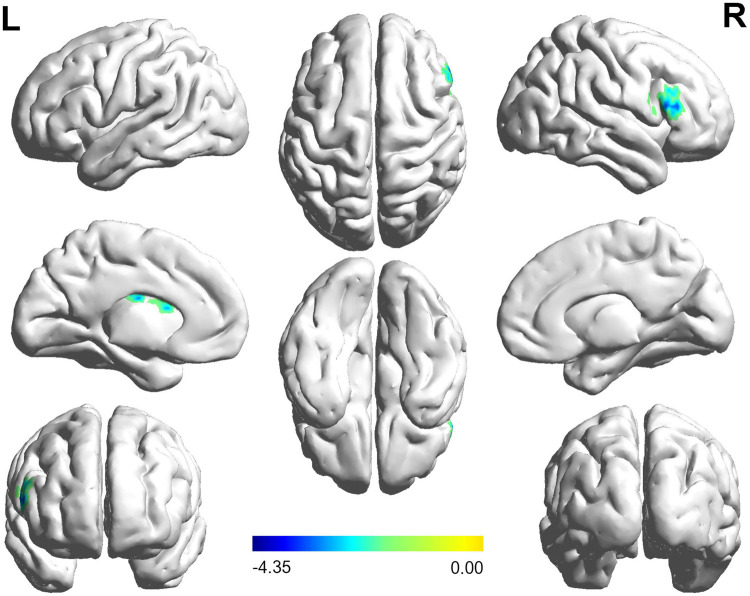
The brain regions with significant FC changes in NAION patients when the left BA17 was used as the seed region. FC, functional connectivity; NAION, non-arteritic anterior ischemic optic neuropathy; BA, Brodmann’s area.

**FIGURE 2:**
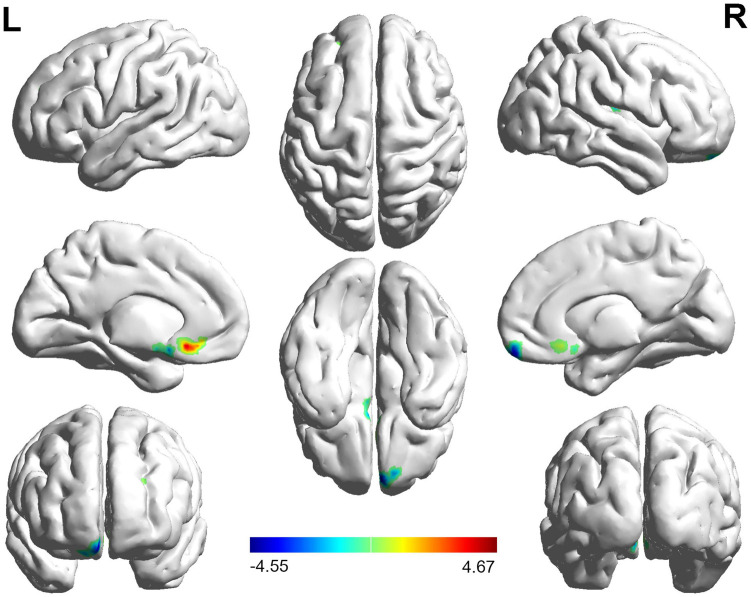
The brain regions with significant FC changes in NAION patients when the right BA17 was used as the seed region. FC, functional connectivity; NAION, non-arteritic anterior ischemic optic neuropathy; BA, Brodmann’s area.

The FC distribution maps for each group are shown in Figures 1, 2 for the left and right BA17, respectively. Compared with HCs, patients with NAION exhibited significantly decreased FC between the left BA17 and the right inferior frontal gyrus (IFG), and left caudate nucleus (Figure 1 and Table 2). Moreover, patients also showed significantly increased FC between the right BA17 and the left olfactory gyrus and significantly decreased FC between the right BA17 and the right SFG, and right insula (Figure 2 and Table 2).

**TABLE 2:** The brain regions with statistically different FC values between the NAION patients and HCs.

**Brain region**	**Hemisphere**	**Peak MNI (mm)**			**Peak *T*-value**	**Cluster size**
		**x**	**y**	**z**		
**Left BA17 as ROI**						
IFG	R	63	15	21	–4.38	28
Caudate nucleus	L	–12	–9	21	–3.97	22
**Right BA17 as ROI**						
SFG	R	6	60	–18	–4.55	16
Olfactory gyrus	L	–3	24	–12	4.67	8
Insula	R	42	–15	15	–3.28	18

*Two-sample t-test was used to determine the differences between NAION patients and HCs. The threshold was set with P < 0.05, corrected for multiple comparisons by the false discovery rate.*

*FC, functional connectivity; NAION, non-arteritic anterior ischemic optic neuropathy; HCs, healthy controls; MNI, Montreal Neurological Institute; BA, Brodmann’s area; ROI, region of interest; IFG, inferior frontal gyrus; SFG, superior frontal gyrus; L, left; R, right.*

### Correlations Between the Clinical Data and Brain Functional Changes in Non-arteritic Anterior Ischemic Optic Neuropathy Patients

**FIGURE 3:**
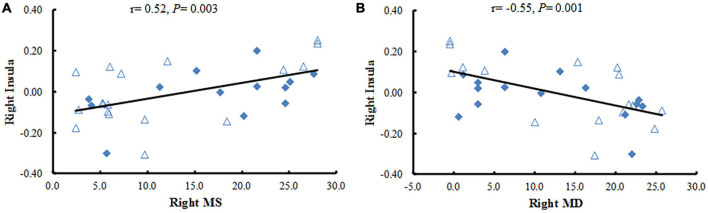
Correlation analysis between the FC values in brain regions with significant differences and clinical variables. **(A)** The Pearson correlation shows a positive association between the mean FC values in the right insula and the right side of MS in NAION patients. **(B)** The Pearson correlation reveals a negative association between the mean FC values in the right insula and the right MD in NAION patients. FC, functional connectivity; NAION, non-arteritic anterior ischemic optic neuropathy; MS, mean sensitivity; MD, mean defect.

In the study, we calculated Pearson correlation coefficients between the mean FC values of the brain regions with statistical difference and the clinical data in patients with NAION. The decreased FC values in the right insula were positively correlated with the right side of mean sensitivity (MS) in the central field of vision (*r* = 0.52, *P* < 0.01) and negatively correlated with the right side of mean defect (MD) in the central field of visual (*r* = −0.55, *P* < 0.01) (Figure 3) when the ROI was the right BA17. No significant correlations were found between mean FC values of any brain regions and the gender, duration of illness, vision acuity, intraocular pressure, or the thickness of the retinal nerve-fiber layer (all *P* ≥ 0.01).

### The Brain Functional Changes in Non-arteritic Anterior Ischemic Optic Neuropathy Patients as Diagnostic Indicators

**FIGURE 4:**
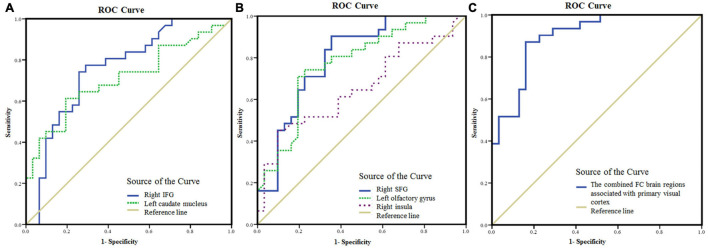
The mean FC values in the brain areas with significant differences between NAION patients and HCs as diagnostic indicators. **(A)** ROC curve analysis of the mean FC values in the right IFG and left caudate nucleus, respectively, for differentiating NAION patients from HCs when the left BA17 was used as the seed region. **(B)** ROC curve analysis of the mean FC values in the right SFG and left olfactory gyrus, respectively, for differentiating NAION patients from HCs when the right BA17 was used as the seed region. **(C)** ROC curve analysis of the combination of the four brain areas (including the right IFG, right SFG, left olfactory gyrus, and left caudate nucleus) for differentiating NAION patients from HCs. FC, functional connectivity; NAION, non-arteritic anterior ischemic optic neuropathy; HCs, healthy controls; BA, Brodmann’s area; IFG, inferior frontal gyrus; SFG, superior frontal gyrus; ROC, receiver operating characteristic.

The areas under the curve (AUC) of FC values in brain regions with statistical difference were as follows (Figure 4): right IFG (0.76, *P* < 0.001, 95% confidence interval (CI): 0.64–0.88), left caudate nucleus (0.71, *P* < 0.001, 95% CI: 0.58–0.84), right SFG (0.80, *P* < 0.001, 95% CI: 0.69–0.91), left olfactory gyrus (0.77, *P* < 0.001, 95% CI: 0.66–0.89), right insula (0.66, *P* < 0.05, 95% CI: 0.52–0.79). The AUC of the FC values in the brain regions associated with the primary visual cortex (including right IFG, right SFG, left olfactory gyrus, and left caudate nucleus) was 0.90 (*P* < 0.001, 95% CI: 0.82–0.97).

## Discussion

In this study, applied seed-based FC analysis, we found significant FC changes in NAION patients compared with HCs. When the left BA17 was the seed region, we found that FC with the right IFG, left caudate nucleus was decreased in patients with NAION. In contrast, when the right BA17 was the seed region, we observed that FC with the right SFG, right insula was decreased in the patients, but that FC with the left olfactory gyrus was increased.

The frontal lobe, located anterior to the central sulcus and above the lateral fissure, is the most complex part of the brain. The IFG has been associated with emotional and cognitive empathy (Shamay-Tsoory et al., 2009), offer quality (de Berker et al., 2019), and attentional control (Hampshire et al., 2010). Previous studies have found that a number of optic disease lead to the IFG dysfunction, including optic neuritis (Sun et al., 2020), anisometropic amblyopia (Lin et al., 2012), strabismus and amblyopia (Shao et al., 2019), and primary angle-closure glaucoma (Chen et al., 2019). Moreover, in a previous study, our team found decreased amplitude of low-frequency fluctuation (ALFF) in the right IFG of patients with NAION (Guo et al., 2019). We hypothesize that these results may reflect the loss of eye motion, reduced cognition, and ongoing dysfunction in the neural networks of these patients. Because of these deficits, patients receive poor visual information about what they are seeing. In support of this theory, the present study found decreased FC between the left BA17 and the right IFG in patients with NAION. In addition, the loss of visual input from the eye diminished activity in corresponding parts of the visual cortex. A previous study has demonstrated that patients with NAION exhibit reduced activation in the occipital cortex when stimulating the affected eye (Aguirregomozcorta et al., 2011). Thus, the decreased FC between BA17 and IFG might reflect compensatory inhibition in patients with NAION that reduces the influence of the poor visual information. In addition to the IFG, many studies have also shown that optic diseases are associated with dysfunction in the SFG (Min et al., 2018; Xu et al., 2019; Wang et al., 2021). The SFG occupies one-third of the frontal lobe and is thought to be the main premotor area (Peng et al., 2021). It plays roles in working memory presentation of visual space (Leavitt et al., 2018), and is also related to acute social stress (Chang and Yu, 2019), cognitive control (Tully et al., 2014), and self-consciousness (Hsieh et al., 2011). One study reported increased ALFF in the right SFG of patients with strabismus and amblyopia (Min et al., 2018). Xu et al. (2019) observed that patients with corneal ulcer demonstrated significantly increased regional homogeneity values in bilateral SFG. The abnormal activity in the SFG might reflect a strengthening of networks in patients with visual loss. Interesting, decreased FC between the BA17 and the right SFG was observed in patients with high-tension glaucoma (Wang et al., 2021). In the present study, we found that the FC values between the right BA17 and the right SFG was decreased in patients. This may indicate an impaired functional network between the SFG and the primary visual areas. Though lacking a detailed statistical analysis, we can speculate that the decreased FC can partially explain the unusual mental state reported in several patients with NAION. In addition, we speculate that NAION might influence brain executive functions and the functional integration of visual information.

The insula plays a critical role in emotion processing (Paulus et al., 2005). Additionally, it is involved in the feeling of anxiety (Paulus and Stein, 2006), as well as threat recognition and conscious urges (Craig, 2002; Lerner et al., 2009). Abnormal brain activity in the insula is also associated with diseases of the eyes, such as glaucoma (Chen et al., 2019), monocular blindness (Shao et al., 2018), and optic neuritis (Shao et al., 2015). Shao et al. (2018) found that patients with monocular blindness in the left eye showed increased voxel-mirrored homotopic connectivity in the insula. However, our results in the current study showed that the FC values between the right BA17 and the right insula was decreased in patients with NAION, which is contrary to what other studies have found (Shao et al., 2015, 2018; Chen et al., 2019). Our new results provide further support for our previous finding that ALFF in patients with NAION is abnormal (Guo et al., 2019). NAION is an acute clinical symptom. It is characterized by sudden, painless unilateral loss of vision, which easily sparks emotional reactions. However, many patients had experienced NAION for several years (average duration, 6.00 ± 1.12 years) before visiting our hospital, and they might have become used to their condition. Thus, the decreased FC with the insula that we observed might reflect an inhibitory effort in patients with NAION to suppress their strong emotions. Meanwhile, we found that the FC values between the right insula and the right BA17 were positively correlated with right side of MS (*r* = 0.52, *P* < 0.01) and negatively correlated with right side of MD (*r* = −0.55, *P* < 0.01). Both MS and MD are key components of the central field of vision, and are important clinical parameters for assessing the severity of ophthalmological lesions; smaller MS and larger MD indicate more severe the damage. Thus, our current results may reflect ongoing damage and the severity in NAION. Furthermore, these correlations may suggest that the severity of ipsilateral damage in the eye extends down through visual-associated cortex. Thus, the more damage is observed in MS and MD, the more we can assume dysfunction in the ipsilateral insula.

The caudate nucleus is a part of the basal ganglia that is involved in a range of functions. The nucleus is thought to play an important role in the regulation of cortical excitability and sensory processing (Villablanca, 2010). Furthermore, the nucleus has afferent, efferent, and loop connectivity with the anterior insula cortex and orbitofrontal gyrus (McGeorge and Faull, 1989). Cai et al. (2015) found that patients with primary angle-closure glaucoma demonstrated increased degree centrality in the left anterior cingulate cortex and caudate. They thought that this finding was related to altered proprioception and somatosensory processing. In addition, the caudate nucleus also plays an important role in processing spatial visual information (Gombköto et al., 2011). Our current results showed that the FC values between the left BA17 and the left caudate nucleus was decreased in the patients with NAION. These results may provide direct evidence that NAION is associated with dysfunction of the caudate nucleus. We think that the decreased FC might reflect neural plasticity that compensates for NAION-related deficits and helps prevent secondary damage.

Interestingly, in addition to the decreased FC reported above, we also found increased FC between the right BA17 and the left olfactory gyrus in the patients with NAION. To compensate for the lack of vision, individuals with early onset blindness often experience enhancements in their remaining senses, including the sense of smell. At the same time, the visual cortex can undergo remodeling so that it can receive and process non-visual inputs (Araneda et al., 2016). Gagnon et al. (2015) found that congenitally blind individuals demonstrated enhanced olfaction compared with sighted controls. The enhanced olfactory function can also develop in optically related diseases. Gugleta et al. (2010) found that patients with primary open-angle glaucoma showed alterations in olfaction. Although the pathogenesis is different, the visual impairment in patients with NAION or primary open-angle glaucoma is secondary. Indeed, research into whether the disease leads to olfactory disorders is lacking. Therefore, our current finding could represent an instance of brain plasticity in which the pathway between the visual cortex and olfactory cortex are strengthened. Thus, we speculate that the increased FC between the right BA17 and the left olfactory gyrus may be a compensatory response to the impaired vision in NAION.

In previous studies (Zhu et al., 2018; Jiang et al., 2019; Su et al., 2020), ROC analyses were successfully used to discriminate ocular disease from HCs. In the present study, ROC analysis was applied to identify patients with NAION. The AUC denotes a relatively good accuracy at values over 0.80. Our results indicated a moderate ability to discriminate patients with NAION from controls using the FC values in the brain areas with statistical difference. Although some regions, including the right IFG, right SFG, left olfactory gyrus, and left caudate nucleus, are located in the non-visual cortex, they functionally connect with the primary visual cortex. Therefore, these regions were visually relevant areas. The AUC value was 0.90 when the above regions were combined. Therefore, the results in the present study indicate that the combination of FC values in these regions may serve as a potential biomarker for distinguishing patients with NAION from HCs.

This study has several limitations. First, neuropsychological tests were not performed in the present study. This was because NAION can be accompanied by strong emotional states in some patients, which might influence the accuracy of the tests. Second, the present study included patients in whom both eyes were affected, but at different times. It is difficult to recruit patients who have only one affected eye because follow-up can occur years after the first eye is affected. Further research is required to examine this issue in more detail. Third, the number of NAION patients in the study was small. The accuracy of the results would be improved with larger sample sizes in future studies.

## Conclusion

Patients with NAION showed significant changes in functional connections between the primary visual cortex and several other brain regions not directly related to visual function. The FC changes in these areas shed light on the neural plasticity in NAION patients and could act as a possible biomarker for distinguishing patients with NAION from HCs. These findings support that NAION may be a neuro-ophthalmological disease.

## Data Availability Statement

The original contributions presented in the study are included in the article/supplementary material, further inquiries can be directed to the corresponding author/s.

## Ethics Statement

The studies involving human participants were reviewed and approved by the Medical Research Ethics Committee and Institutional Review Board of Dong Fang Hospital affiliated with Beijing University of Chinese Medicine, Beijing, China (No. JDF-IRB-2015031102). The patients/participants provided their written informed consent to participate in this study. Written informed consent was obtained from the individual(s) for the publication of any potentially identifiable images or data included in this article.

## Author Contributions

PG conceived the study, participated in the design, and wrote most of the manuscript. PZ and YS recruited patients and collected their clinical data. HL analyzed and interpreted the data. ML scanned the participants. YW, HH, and SK organized the database and carried out the statistical analysis. All authors read and approved the final manuscript.

## Conflict of Interest

The authors declare that the research was conducted in the absence of any commercial or financial relationships that could be construed as a potential conflict of interest.

## Publisher’s Note

All claims expressed in this article are solely those of the authors and do not necessarily represent those of their affiliated organizations, or those of the publisher, the editors and the reviewers. Any product that may be evaluated in this article, or claim that may be made by its manufacturer, is not guaranteed or endorsed by the publisher.

## Footnotes

1
http://www.rfmri.org/DPARSF_V2_3

2
https://www.fil.ion.ucl.ac.uk/spm/

3
http://www.restfmri.net/forum/REST_V1.8

4
https://www.nitrc.org/projects/bnv/

## References

[B1] Aguirregomozcorta M. Mancini L. Jenkins T. M. Hickman S. J. Ciccarelli O. Plant G. T. (2011). A longitudinal functional MRI study of non-arteritic anterior ischaemic optic neuropathy patients. *J. Neurol. Neurosurg. Psychiatry* 82 905–913. 10.1136/jnnp.2009.194563 21285455

[B2] Araneda R. Renier L. A. Rombaux P. Cuevas I. De Volder A. G. (2016). Cortical plasticity and olfactory function in early blindness. *Front. Syst. Neurosci.* 10:75. 10.3389/fnsys.2016.00075 27625596 PMC5003898

[B3] Berry S. Lin W. V. Sadaka A. Lee A. G. (2017). Nonarteritic anterior ischemic optic neuropathy: cause, effect, and management. *Eye Brain* 9 23–28. 10.2147/EB.S125311 29033621 PMC5628702

[B4] Burton H. Sinclair R. J. McLaren D. G. (2004). Cortical activity to vibrotactile stimulation: an fMRI study in blind and sighted individuals. *Hum. Brain Mapp.* 23 210–228. 10.1002/hbm.20064 15449356 PMC3697024

[B5] Cai F. Gao L. Gong H. Jiang F. Pei C. Zhang X. (2015). Network centrality of resting-state fMRI in primary angle-closure glaucoma before and after surgery. *PLoS One* 10:e0141389. 10.1371/journal.pone.0141389 26506229 PMC4624709

[B6] Chang J. Yu R. (2019). Acute social stress modulates coherence regional homogeneity. *Brain Imaging Behav.* 13 762–770. 10.1007/s11682-018-9898-9 29802600

[B7] Chen L. Li S. Cai F. Wu L. Gong H. Pei C. (2019). Altered functional connectivity density in primary angle-closure glaucoma patients at resting-state. *Quant. Imaging Med. Surg.* 9 603–614. 10.21037/qims.2019.04.13 31143651 PMC6511722

[B8] Craig A. D. (2002). How do you feel? Interoception: the sense of the physiological condition of the body. *Nat. Rev. Neurosci.* 3 655–666. 10.1038/nrn894 12154366

[B9] Dai P. Zhang J. Wu J. Chen Z. Zou B. Wu Y. (2019). Altered spontaneous brain activity of children with unilateral amblyopia: a resting state fMRI study. *Neural Plast.* 2019:3681430. 10.1155/2019/3681430 31428144 PMC6683781

[B10] de Berker A. O. Kurth-Nelson Z. Rutledge R. B. Bestmann S. Dolan R. J. (2019). Computing value from quality and quantity in human decision-making. *J. Neurosci.* 39 163–176. 10.1523/JNEUROSCI.0706-18.2018 30455186 PMC6325261

[B11] Ding K. Liu Y. Yan X. Lin X. Jiang T. (2013). Altered functional connectivity of the primary visual cortex in subjects with amblyopia. *Neural Plast.* 2013:612086. 10.1155/2013/612086 23844297 PMC3697400

[B12] Gagnon L. Ismaili A. R. Ptito M. Kupers R. (2015). Superior orthonasal but not retronasal olfactory skills in congenital blindness. *PLoS One* 10:e0122567. 10.1371/journal.pone.0122567 25822780 PMC4379017

[B13] Gombköto P. Rokszin A. Berényi A. Braunitzer G. Utassy G. Benedek G. (2011). Neuronal code of spatial visual information in the caudate nucleus. *Neuroscience* 182 225–231. 10.1016/j.neuroscience.2011.02.048 21376107

[B14] Gugleta K. Kochkorov A. Katamay R. Husner A. Welge-Lüssen A. Flammer J. (2010). Olfactory function in primary open-angle glaucoma patients. *Klin. Monbl. Augenheilkd.* 227 277–279. 10.1055/s-0029-1245198 20408073

[B15] Guo P. D. Zhao P. B. Lv H. Man F. Y. Su Y. Zhao J. (2019). Abnormal spontaneous brain activity in patients with non-arteritic anterior ischemic optic neuropathy detected using functional magnetic resonance imaging. *Chin. Med. J.* 132 741–743. 10.1097/CM9.0000000000000134 30855358 PMC6416029

[B16] Guo P. Zhao P. Lv H. Su Y. Liu M. Chen Y. (2020). Abnormal regional spontaneous neural activity in nonarteritic anterior ischemic optic neuropathy: a resting-state functional MRI study. *Neural Plast.* 2020:8826787. 10.1155/2020/8826787 32963518 PMC7499295

[B17] Hampshire A. Chamberlain S. R. Monti M. M. Duncan J. Owen A. M. (2010). The role of the right inferior frontal gyrus: inhibition and attentional control. *Neuroimage* 50 1313–1319. 10.1016/j.neuroimage.2009.12.109 20056157 PMC2845804

[B18] Hattenhauer M. G. Leavitt J. A. Hodge D. O. Grill R. Gray D. T. (1997). Incidence of nonarteritic anterior ischemic optic neuropathy. *Am. J. Ophthalmol.* 123:103. 10.1016/S0002-9394(14)70999-7 9186104

[B19] Hsieh C. W. Wu J. H. Hsieh C. H. Wang Q. F. Chen J. H. (2011). Different brain network activations induced by modulation and nonmodulation laser acupuncture. *Evid. Based Complement Alternat. Med.* 2011:951258. 10.1155/2011/951258 20953400 PMC2952336

[B20] Huang X. Zhou F. Q. Dan H. D. Shen Y. (2018). Abnormal intrinsic brain activity in individuals with peripheral vision loss because of retinitis pigmentosa using amplitude of low-frequency fluctuations. *Neuroreport* 29 1323–1332. 10.1097/WNR.0000000000001116 30113921

[B21] Jiang F. Yu C. Zuo M. J. Zhang C. Wang Y. Zhou F. Q. (2019). Frequency-dependent neural activity in primary angle-closure glaucoma. *Neuropsychiatr. Dis. Treat.* 15 271–282. 10.2147/NDT.S187367 30697052 PMC6342137

[B22] Johnson L. N. Arnold A. C. (1994). Incidence of nonarteritic and arteritic anterior ischemic optic neuropathy. population-based study in the state of Missouri and Los Angeles County, California. *J. Neuroophthalmol.* 14 38–44. 10.1097/00041327-199403000-00011 8032479

[B23] Katz D. M. Trobe J. D. (2015). Is there treatment for nonarteritic anterior ischemic optic neuropathy. *Curr. Opin. Ophthalmol.* 26 458–463. 10.1097/ICU.0000000000000199 26367094

[B24] Keren S. Zanolli M. Dotan G. (2017). Visual outcome following bilateral non-arteritic anterior ischemic optic neuropathy: a systematic review and meta-analysis. *BMC Ophthalmol.* 17:155. 10.1186/s12886-017-0543-y 28836961 PMC5571512

[B25] Knox D. L. Kerrison J. B. Green W. R. (2000). Histopathologic studies of ischemic optic neuropathy. *Trans. Am. Ophthalmol. Soc.* 98 203–20. 11190024 PMC1298227

[B26] Leavitt M. L. Pieper F. Sachs A. J. Martinez-Trujillo J. C. (2018). A quadrantic bias in prefrontal representation of visual-mnemonic space. *Cereb. Cortex* 28 2405–2421. 10.1093/cercor/bhx142 28605513

[B27] Lerner A. Bagic A. Hanakawa T. Boudreau E. A. Pagan F. Mari Z. (2009). Involvement of insula and cingulate cortices in control and suppression of natural urges. *Cereb. Cortex* 19 218–223. 10.1093/cercor/bhn074 18469316 PMC2638741

[B28] Li Q. Bai J. Zhang J. Gong Q. Liu L. (2016). Assessment of cortical dysfunction in patients with intermittent exotropia: an fMRI study. *PLoS One* 11:e0160806. 10.1371/journal.pone.0160806 27501391 PMC4976854

[B29] Lin X. Ding K. Liu Y. Yan X. Song S. Jiang T. (2012). Altered spontaneous activity in anisometropic amblyopia subjects: revealed by resting-state FMRI. *PLoS One* 7:e43373. 10.1371/journal.pone.0043373 22937041 PMC3427333

[B30] Liu F. Guo W. Fouche J. P. Wang Y. Wang W. Ding J. (2015). Multivariate classification of social anxiety disorder using whole brain functional connectivity. *Brain Struct. Funct.* 220 101–115. 10.1007/s00429-013-0641-4 24072164

[B31] Liu F. Wang Y. Li M. Wang W. Li R. Zhang Z. (2017). Dynamic functional network connectivity in idiopathic generalized epilepsy with generalized tonic-clonic seizure. *Hum. Brain Mapp.* 38 957–973. 10.1002/hbm.23430 27726245 PMC6866949

[B32] Lv H. Wang Z. Tong E. Williams L. M. Zaharchuk G. Zeineh M. (2018). Resting-state functional MRI: everything that nonexperts have always wanted to know. *Am. J. Neuroradiol.* 39 1390–1399. 10.3174/ajnr.A5527 29348136 PMC6051935

[B33] Maldjian J. A. Laurienti P. J. Burdette J. H. (2004). Precentral gyrus discrepancy in electronic versions of the talairach atlas. *Neuroimage* 21 450–455. 10.1016/j.neuroimage.2003.09.032 14741682

[B34] Maldjian J. A. Laurienti P. J. Kraft R. A. Burdette J. H. (2003). An automated method for neuroanatomic and cytoarchitectonic atlas-based interrogation of fMRI data sets. *Neuroimage* 19 1233–1239. 10.1016/s1053-8119(03)00169-1 12880848

[B35] McGeorge A. J. Faull R. L. (1989). The organization of the projection from the cerebral cortex to the striatum in the rat. *Neuroscience* 29 503–537. 10.1016/0306-4522(89)90128-0 2472578

[B36] Miller N. R. Arnold A. C. (2015). Current concepts in the diagnosis, pathogenesis and management of nonarteritic anterior ischaemic optic neuropathy. *Eye* 29 65–79. 10.1038/eye.2014.144 24993324 PMC4289822

[B37] Min Y. L. Su T. Shu Y. Q. Liu W. F. Chen L. L. Shi W. Q. (2018). Altered spontaneous brain activity patterns in strabismus with amblyopia patients using amplitude of low-frequency fluctuation: a resting-state fMRI study. *Neuropsychiatr. Dis. Treat.* 14 2351–2359. 10.2147/NDT.S171462 30275692 PMC6157537

[B38] Paulus M. P. Stein M. B. (2006). An insular view of anxiety. *Biol. Psychiatry* 60 383–387. 10.1016/j.biopsych.2006.03.042 16780813

[B39] Paulus M. P. Feinstein J. S. Castillo G. Simmons A. N. Stein M. B. (2005). Dose-dependent decrease of activation in bilateral amygdala and insula by lorazepam during emotion processing. *Arch. Gen. Psychiatry* 62 282–288. 10.1001/archpsyc.62.3.282 15753241

[B40] Peng Z. Y. Liu Y. X. Li B. Ge Q. M. Liang R. B. Li Q. Y. (2021). Altered spontaneous brain activity patterns in patients with neovascular glaucoma using amplitude of low-frequency fluctuations: a functional magnetic resonance imaging study. *Brain Behav.* 11:e02018. 10.1002/brb3.2018 33386699 PMC7994689

[B41] Qin Y. Yuan W. Deng H. Xiang Z. Yang C. Kou X. (2015). Clinical efficacy observation of acupuncture treatment for nonarteritic anterior ischemic optic neuropathy. *Evid. Based Complement Alternat. Med.* 2015:713218. 10.1155/2015/713218 26089945 PMC4458289

[B42] Shamay-Tsoory S. G. Aharon-Peretz J. Perry D. (2009). Two systems for empathy: a double dissociation between emotional and cognitive empathy in inferior frontal gyrus versus ventromedial prefrontal lesions. *Brain* 132 617–627. 10.1093/brain/awn279 18971202

[B43] Shao Y. Bao J. Huang X. Zhou F. Q. Ye L. Min Y. L. (2018). Comparative study of interhemispheric functional connectivity in left eye monocular blindness versus right eye monocular blindness: a resting-state functional MRI study. *Oncotarget* 9 14285–14295. 10.18632/oncotarget.24487 29581843 PMC5865669

[B44] Shao Y. Cai F. Q. Zhong Y. L. Huang X. Zhang Y. Hu P.-H. (2015). Altered intrinsic regional spontaneous brain activity in patients with optic neuritis: a resting-state functional magnetic resonance imaging study. *Neuropsychiatr. Dis. Treat.* 11 3065–3073. 10.2147/NDT.S92968 26715848 PMC4686319

[B45] Shao Y. Li Q. H. Li B. Lin Q. Su T. Shi W. Q. (2019). Altered brain activity in patients with strabismus and amblyopia detected by analysis of regional homogeneity: a resting-state functional magnetic resonance imaging study. *Mol. Med. Rep.* 19 4832–4840. 10.3892/mmr.2019.10147 31059016 PMC6522834

[B46] Su T. Yuan Q. Liao X. L. Shi W. Q. Zhou X. Z. Lin Q. (2020). Altered intrinsic functional connectivity of the primary visual cortex in patients with retinal vein occlusion: a resting-state fMRI study. *Quant. Imaging Med. Surg.* 10 958–969. 10.21037/qims.2020.03.24 32489920 PMC7242310

[B47] Sun M. Zhou H. Xu Q. Yang M. Xu X. Zhou M. (2020). Differential patterns of interhemispheric functional connectivity between AQP4-optic neuritis and MOG-optic neuritis: a resting-state functional MRI study. *Acta Radiol.* 62 776–783. 10.1177/0284185120940250 32660318

[B48] Tononi G. Sporns O. Edelman G. M. (1994). A measure for brain complexity: relating functional segregation and integration in the nervous system. *Proc. Natl. Acad. Sci. U.S.A.* 91 5033–5037. 10.1073/pnas.91.11.5033 8197179 PMC43925

[B49] Tully L. M. Lincoln S. H. Liyanage-Don N. Hooker C. I. (2014). Impaired cognitive control mediates the relationship between cortical thickness of the superior frontal gyrus and role functioning in schizophrenia. *Schizophr. Res.* 152 358–364. 10.1016/j.schres.2013.12.005 24388000

[B50] van Kemenade B. M. Arikan B. E. Kircher T. Straube B. (2017). The angular gyrus is a supramodal comparator area in action-outcome monitoring. *Brain Struct. Funct.* 222 3691–3703. 10.1007/s00429-017-1428-9 28439662

[B51] Villablanca J. R. (2010). Why do we have a caudate nucleus. *Acta Neurobiol. Exp.* 70 95–105. 10.55782/ane-2010-1778 20407491

[B52] Wang B. Yan T. Zhou J. Xie Y. Qiu J. Wang Y. (2021). Altered fMRI-derived functional connectivity in patients with high-tension glaucoma. *J. Neuroradiol.* 48 94–98. 10.1016/j.neurad.2020.03.001 32169470

[B53] Wang K. Jiang T. Yu C. Tian L. Li J. Liu Y. (2008). Spontaneous activity associated with primary visual cortex: a resting-state FMRI study. *Cereb. Cortex* 18 697–704. 10.1093/cercor/bhm105 17602140

[B54] Wang M. Y. Qi P. H. Shi D. P. (2011). Diffusion tensor imaging of the optic nerve in subacute anterior ischemic optic neuropathy at 3T. *Am. J. Neuroradiol.* 32 1188–1194. 10.3174/ajnr.A2487 21700789 PMC7966056

[B55] Xu L. Wang Y. Jonas J. B. (2007). Incidence of nonarteritic anterior ischemic optic neuropathy in adult Chinese: the Beijing eye study. *Eur J Ophthalmol.* 17 459–460. 10.1177/112067210701700335 17534837

[B56] Xu M. W. Liu H. M. Tan G. Su T. Xiang C. Q. Wu W. (2019). Altered regional homogeneity in patients with corneal ulcer: a resting-state functional MRI study. *Front. Neurosci.* 13:743. 10.3389/fnins.2019.00743 31396034 PMC6664059

[B57] Zhang C. Guo Y. Slater B. J. Miller N. R. Bernstein S. L. (2010). Axonal degeneration, regeneration and ganglion cell death in a rodent model of anterior ischemic optic neuropathy (rAION). *Exp. Eye Res.* 91 286–292. 10.1016/j.exer.2010.05.021 20621651 PMC2907443

[B58] Zhu P. W. Huang X. Ye L. Jiang N. Zhong Y. L. Yuan Q. (2018). Altered intrinsic functional connectivity of the primary visual cortex in youth patients with comitant exotropia: a resting state fMRI study. *Int. J. Ophthalmol.* 11 668–673. 29675389 10.18240/ijo.2018.04.22 PMC5902375

